# Interparental Conflict and Korean Children’s Inhibitory Control: Testing Emotional Insecurity as a Mediator

**DOI:** 10.3389/fpsyg.2021.632052

**Published:** 2021-06-14

**Authors:** Young-Eun Lee, SoJung Seo

**Affiliations:** ^1^Department of Early Childhood Education, Gachon University, Seongnam, South Korea; ^2^Department of Child & Family Studies, Kyung Hee University, Seoul, South Korea

**Keywords:** interparental conflict, inhibitory control, emotional insecurity, involvement, emotional reactivity, avoidance

## Abstract

This study examined the association between interparental conflict and inhibitory control of Korean children, and it investigated whether this relationship is mediated by the single latent variable of emotional insecurity or by three dimensions of emotional insecurity (i.e., involvement, emotional reactivity, and avoidance). A total of 166 mother–father dyads with Korean children aged 3–5 years participated in a short-term longitudinal survey at two-time points. Both parents completed measures of interparental conflict and emotional insecurity, and, 6 months later, they completed a measure of the inhibitory control of children. The structural equation modeling results suggested that interparental conflict was related to poor inhibitory control in Korean children. Emotional insecurity did not mediate the association between interparental conflict and inhibitory control. Of the three emotional insecurity dimensions, the involvement partially mediated the relationship. Interestingly, although interparental conflict predicted poor inhibitory control, the involvement of children in the conflict, which was associated with a greater interparental conflict, predicted a subsequent increase in inhibitory control 6 months later. These findings were interpreted in terms of sensory processing patterns, the reformulation of emotional security theory, and the influence of Confucianism on Korean culture.

## Introduction

The adjustment problems in children (i.e., externalizing and internalizing behaviors) are the most consistent outcomes when emotional security is threatened by interparental conflict (Cummings and Davies, 2010). Although the links between interparental conflict, emotional security of children, and adjustment problems in children are well established, less is known about how the concerns of children for their safety or a threatened sense of security increase their vulnerability to adjustment problems (Davies et al., 2013). Thus, more research is needed that focuses on the psychological functions of children, whose functions are affected by emotional insecurity and which, in turn, leads to adjustment problems.

Inhibitory control, which is a specific aspect of the executive functioning of the cognitive system, is the ability to suppress prepotent and automatic thoughts or actions through internally represented goals (Miller and Cohen, 2001; Diamond, 2013). It prevents developmental maladaptive outcomes by overriding internal tendencies and external stimuli and regulating impulsive desire. Prior research studies have revealed that inhibitory control is longitudinally associated with fewer externalizing problems and psychosocial adversity (Olson et al., 2011; Buss et al., 2014). It is also a significant longitudinal predictor of better emotion regulation, stronger conscience, greater committed compliance, socioemotional competence, attention, and academic performance (Kochanska et al., 2000, 2001; Kochanska and Knaack, 2003; Rhoades et al., 2009; Jaekel et al., 2016). However, few studies have examined how emotional security of children is being threatened by interparental conflict affecting inhibitory control. Moreover, existing studies of emotional security processes in children have focused on white families in the United States (Davies et al., 2016a,b).

Therefore, the first aim of this study was to examine whether interparental conflict predicts inhibitory control of children. A substantial number of studies on inhibitory control have regarded it as a temperamental attribute and focused on its moderating role in the association between the environment and the developmental outcomes in the child (Lengua et al., 2008; Thompson et al., 2020). This research trend is also exhibited in studies conducted in Asian cultures. For example, in South Korea, inhibitory/effortful control has been found to moderate the relationship between marital conflicts and problem behaviors of preschoolers (Moon and Park, 2020). However, inhibitory control, which is considered a temperamental self-regulatory trait, can also be directly affected by interparental conflict. According to social learning theory, when children are exposed to a hostile interparental conflict, they observe and model destructive behavior of their parents, reduce their inhibition of aggression, and increase impulsive and angry responses (Emery, 1989; Davies and Sturge-Apple, 2014). Changes in inhibitory control can also occur through the socialization practices of parents, although inhibitory control appears early and has relatively stable behavioral properties (Rothbart, 1989; Eisenberg et al., 2004). Recently, Xing et al. (2019) revealed that inhibitory control of Chinese children was undermined by harsh parental discipline but increased by parental warmth. In Asian and Western studies that were based on family systems theory, the interparental conflict has emerged as a significant direct predictor of parental socialization (Lee, 2018; Lee and Brophy-Herb, 2018). Thus, this study examined the direct effect of interparental conflict on inhibitory control of children.

The second aim of this study was to investigate whether emotional insecurity mediates the relationship between interparental conflict and inhibitory control. According to emotional security theory (EST), destructive conflicts between parents increase the vulnerability of children to adjustment to problems by elevating their emotional insecurity (Davies and Cummings, 1994). Repeated exposure to interparental conflict, including uncooperative attitudes, hostile interactions, and neglect, can be a toxic environment that undermines the goal of children of preserving their safety and security. The concerns of children about security cannot be observed directly, but they can be inferred from involvement (e.g., mediating the argument, comforting parents), negative emotional reactions (e.g., appearing frightened, sad, or upset), and avoidance (e.g., escaping the argument) of interparental conflict (Davies et al., 2002a).

In this study, we hypothesized that emotional insecurity would act as a mediator when interparental conflict reduces inhibitory control of children. Our hypothesis was based on the regulatory depletion model proposed by Muraven and Baumeister (2000). This model proposes that performing regulation functions in one situation (e.g., the process of preserving emotional security) depletes the capacity to effectively regulate another situation (e.g., an inhibitory control task). In other words, children who are distressed by interparental discord may drain their self-regulatory resources to preserve their emotional security. If children exhaust their regulatory resources as the conflicts worsen, regulatory depletion can manifest as an expression of emotional insecurity. In turn, they are unable to engage in regulatory activities, such as inhibitory control tasks.

By applying the regulatory depletion model, Davies et al. (2013) proposed that the emotional insecurity of children occurring during interparental discord might amplify their distress and vigilance and limit their chances of developing successful strategies to regulate emotions. The authors found that the emotional insecurity of children influenced their success in resolving stage-salient tasks an year later. It was suggested that children who are exposed to the interparental conflict could restrict their problem-solving ability because the successful use and integration of internal and external resources were limited by decreased frustration tolerance. Additionally, the authors argued that difficulties of children with problem-solving could further impair the executive functions necessary for controlling impulses (e.g., inhibitory control). Martin et al. (2017) also demonstrated that insecure representations of the interparental relationship of children predicted their problems with executive functions, which refer to purposeful skills, including inhibitory control. The authors interpreted the result as supporting the regulatory depletion model, in which emotional insecurity of children can lead to the sacrifice of their emotional and informational processing capabilities by prioritizing self-defense while identifying and responding to threats in this parental subsystem.

The current study also examined the mediating effects of each dimension of emotional insecurity (i.e., involvement, emotional reactivity, and avoidance) on the association between interparental conflict and inhibitory control for two reasons. First, although most prior research assumed that emotional insecurity was a single negative aspect of response to interparental conflict (Cummings et al., 2012; Davies et al., 2016a), Cummings and Davies (1996) described the emotional security process as a dynamic and nonlinear control system. According to Davies and Martin (2013), the magnitude of the relationship among the multiple indicators of emotional security is modest, and the mean shared variance among the measures of emotional security was found to be only 17% in a review of 15 studies. This may be because the sensory processing patterns differ for each person. Sensory processing refers to the ability to receive, organize, modulate, and interpret sensory stimuli using sensory systems, such as the visual, auditory, and tactile systems, and to respond to the situational demands in everyday life (Miller et al., 2012). Depending on their sensory processing pattern, children may exhibit different emotional insecurity behaviors, and these behaviors may affect their inhibition control differently.

Second, because of the Confucian culture of Korean families, the expressions of emotional insecurity in children in response to the interparental conflict may be more diverse. According to the cognitive-contextual framework (Grych and Fincham, 1990), efforts of children to understand and cope with the conflict as well as with the distal context factors, such as the emotional climate and temperament of family, affect their response to interparental conflict. Korean families are good samples for explaining the role of cultural specificity of Confucianism in the emotional security process. Most studies in this field have tested the concept of EST in Western children and parents, especially those from North American families. However, few Asian studies have investigated the emotional security process based on EST. Today, in Korea, the familism of Confucianism is still strong, although some attributes of Confucianism have diminished (Lee and Bauer, 2013). On the one hand, since familism emphasizes warm, close, interconnected, and supportive family relationships (Campos et al., 2014), Korean children may try to mediate conflicts in the interparental discord context to strengthen parental solidarity or maintain family bonds. On the other hand, the hierarchical relationship of Confucianism still exists in the current Korean family system (Kim and Choi, 2014). In the hierarchical relationship between parents and children, Korean children may have less authority to intervene in parental problems than Western children, or they may avoid interparental conflict situations that undermine the authority of parents. Thus, in the current study, we divided emotional insecurity into three dimensions and examined the mediating effect of each dimension on the association between interparental conflict and inhibitory control, after assessing the mediation effect of emotional insecurity as a single latent factor.

Additionally, this study investigated emotional security and inhibitory control in children aged 3–5 years for the following reasons. First, children under 5 years of age are more exposed to interparental conflict and violence than children of a higher age (Fantuzzo et al., 1997). Second, preschool children experience more fear and threats than older children when exposed to interparental conflict (Kitzmann et al., 2003). Third, although there are individual differences in inhibitory control before the age of 1 (Putnam and Stifter, 2002), it develops rapidly during early childhood, and it can change depending on the environment (Kochanska et al., 1996; Carlson, 2005; Sheese et al., 2008; Xing et al., 2019). Lastly, emotional security processes and the level of inhibitory control experienced during the preschool period may affect psychological adjustment and academic achievement throughout adolescence and young adulthood (Kouros et al., 2010; Walker and Henderson, 2012; Martin et al., 2017).

## Materials and Methods

### Participants

A total of 332 parents (166 mother–father dyads) of preschool-aged children were recruited from daycare centers and preschools in six urban areas of South Korea. Gift cards with the value of $10 were provided to participants who completed the questionnaire at Time 1 and Time 2 (6 months later). All research procedures were approved by the Institutional Review Board at Kyung Hee University prior to conducting the study (approval number: KHSIRB-18-070). The ages of mothers ranged from 22 to 47 years, with a mean of 36.53 (*SD* = 3.39). The ages of fathers ranged from 22 to 52 years, with a mean of 38.34 (*SD* = 4.04). The average age of the children was 59.63 months (*SD* = 12.71). Of these children, 52% (*n* = 86) were boys. All mothers and fathers were married and cohabiting, and they were the biological parents of their children. They were highly educated, with a median education level of a 4-year college degree. Half of the mothers (*n* = 83) were unemployed, whereas a substantial majority of the fathers (98%; *n* = 82) were employed. Approximately 94% of the mothers were primary caregivers. The median monthly family income of the participants was between 4,070,000 ₩ (US$3,409) and 5,400,000 ₩ (US$4,523).

### Measures

#### Interparental Conflict

At Time 1, the interparental conflict was measured using items from the Conflicts and Problem-Solving Scales (CPS; Kerig, 1996), which were translated into Korean and modified for our context of study. Both mothers and fathers independently completed the frequency subscale (one item), which assesses the number of times they were engaged in major conflicts (e.g., “How often do you/your spouse have major disagreements?”). The subscale items were rated using a 6-point scale that ranged from 1 (*once a year or less*) to 6 (*just about every day*). Additionally, mothers and fathers completed the child involvement subscale (five items), which measures the degree of the involvement of children in the conflict (e.g., arguing in front of the child); the verbal aggression subscale (eight items), which assesses the tendency to threaten or inflict harm on a partner in a verbal manner (e.g., cursing); and the cooperation subscale (six items), which captures collaborative efforts to solve the interparental conflict in a respectful way (e.g., listening to the point of view of the spouse). All of these items of subscales were rated using a 4-point scale that ranged from 0 (*never*) to 3 (*often*). The Cronbach’s alpha values for the mothers in this sample ranged from 0.74 to 0.85 (*M* = 0.81), and for the fathers, it ranged from 0.72 to 0.87 (*M* = 0.80). A paired-sample *t*-test was conducted to evaluate the mean differences in the conflict strategies between the mothers and fathers; there were no differences in the frequency subscale (*M* diff = 0.00; *t* = −0.26, *ns*), the verbal aggression subscale (*M* diff = 0.03; *t* = 1.44, *ns*), or the cooperation subscale (*M* diff = 0.00; *t* = 0.00, *ns*). Mothers scored more on the child involvement subscale (*M* diff = 0.08; *t* = 5.17, *p* < 0.01). The reports of mothers and fathers were averaged and standardized, and their scores were found to be significantly correlated (frequency subscale: *r* = 0.32, *p* < 0.01; child involvement subscale: *r* = 0.42, *p* < 0.01; verbal aggression subscale: *r* = 0.34, *p* < 0.01; and cooperation subscale: *r* = 0.30, *p* < 0.01). Therefore, to form a single aggregate score for each dimension of interparental conflict, the scores were summed across couples after the cooperation subscale was reversed (i.e., higher scores indicated uncooperative conflict).

#### Emotional Insecurity About Interparental Conflict

At Time 1, the mothers and fathers completed the Security in the Marital Subsystem-Parent Report (SIMS-PR; Davies et al., 2002a), which was translated into Korean by Lee and Seo (2020). The parents separately completed the involvement subscale (seven items), which captures the attempts of children to intervene in interparental conflict (e.g., trying to help us to solve the problem); the emotional reactivity subscale (nine items), which measures negative expressions of intense and dysregulated distress in children (e.g., appearing frightened); and the avoidance subscale (four items), which assesses the strategies of children to escape or avoid the interparental conflict or its adverse aftermath (e.g., trying to get away from us). The subscale items were rated using a 5-point scale that ranged from 1 (*not at all like him or her*) to 5 (*a whole lot like him or her*). The Cronbach’s alpha values for the mothers in this sample ranged from 0.68 to 0.88 (*M* = 0.81), and for the fathers, it ranged from 0.72 to 0.89 (*M* = 0.83). A paired-sample *t*-test was conducted to examine the mean differences in the emotional insecurity of children between the mothers and fathers; there were no differences in the emotional reactivity subscale (*M* diff = 0.13; *t* = 1.87, *ns*) or the avoidance subscale (*M* diff = 0.08; *t* = −1.42, *ns*). Mothers scored more on the involvement subscale (*M* diff = 0.19; *t* = 2.83, *p* < 0.01). The reports of mothers and fathers were averaged, and their scores were found to be significantly correlated (involvement subscale: *r* = 0.55, *p* < 0.0.1; emotional reactivity subscale: *r* = 0.38, *p* < 0.01; avoidance subscale: *r* = 0.33, *p* < 0.01). Therefore, to create a single aggregate score for each dimension of emotional insecurity, the scores were summed across couples.

#### Inhibitory Control of Child

At Time 2, the mothers and fathers completed the inhibitory control subscale (13 items) of the Child Behavior Questionnaire (Rothbart et al., 1994), which was translated into Korean and verified for reliability and validity by Lee (2004). The parents were asked to report the ability of their children to suppress immediate behavioral reactions or initiate appropriate behavior when directed (e.g., is good at following instructions). All of the items of the subscale were rated using a 7-point scale that ranged from 1 (*extremely untrue of my child*) to 7 (*extremely true of my child*). Negatively worded items were reverse coded so that higher scores represented higher levels of inhibitory control. The Cronbach’s alpha values were 0.81 and 0.83 for the mothers and fathers, respectively. A paired-sample *t*-test was conducted to assess the mean differences in the inhibitory control of children between mothers and fathers; the mothers scored high on the inhibitory control subscale (*M* diff = 0.28; *t* = 4.70, *p* < 0.01). The reports of mothers and fathers were averaged and they were found to be significantly correlated (*r* = 0.43, *p* < 0.0.1). To form a single aggregate score for inhibitory control, the scores were summed across couples.

#### Covariates

The gender of the child (0 = boys and 1 = girls) was included as a covariate in all of the models because extensive previous research into child inhibitory control has considered the gender of the child to be an important control variable (Kochanska et al., 1997; Moilanen et al., 2009; Xing et al., 2019). In our sample, however, there was no mean difference in the inhibitory control of the child according to the gender of the child (*t* = −1.12; *ns*).

## Results

The means, SDs, and correlations of the study variables are shown in Table 1. Regarding the emotional insecurity dimensions, involvement had the highest mean (*M* = 4.77, *SD* = 1.66), whereas avoidance had the lowest mean (*M* = 3.12, *SD* = 0.99). Using the cutoffs of 2 and 7 for skewness and kurtosis, respectively (West et al., 1995), all of the main variables were normally distributed, and no missing data were observed. Structural equation modeling (SEM) was used to test our three hypotheses: (1) that interparental conflict would predict inhibitory control of children 6 months later, (2) that the latent variable of emotional insecurity would mediate the relationship between interparental conflict and inhibitory control, and (3) that each dimension of emotional insecurity (i.e., involvement, emotional reactivity, and avoidance) would individually mediate the relationship between interparental conflict and inhibitory control. The analyses were performed using Mplus 8.3 (Muthén and Muthén, 2017). The maximum likelihood robust estimator was utilized to account for non-normality and non-independence of the data, and the bootstrap method (5,000 bootstrap samples) was employed to test the significance of the mediation/indirect effects. The cutoffs of the comparative fit index (CFI) ≥ 0.95 (Hu and Bentler, 1999), standardized root mean square residual (SRMR) ≤ 0.08 (Hu and Bentler, 1999), root mean square error of approximation (RMSEA) ≤ 0.07 (Steiger, 2007), and a relative *χ*^2^ index (*χ*^2^/*df*) < 3 (Kline, 1998) were considered the criteria for a relatively good fit with the data and hypothesized model.

**Table 1:** Descriptive statistics and correlations between the study variables.

	Minimum	Maximum	*M*	*SD*	1	2	3	4	5	6	7	8
**Time 1 Interparental conflict**												
1. Frequency of conflict	0.00	1.00	0.15	0.25	-							
2. Uncooperative conflict	0.00	1.11	0.25	0.22	0.48^∗∗∗^	-						
3. Child-related conflict	0.00	1.47	0.59	0.30	0.45^∗∗∗^	0.41^∗∗∗^	-					
4. Verbal aggression	0.00	1.59	0.79	0.32	0.46^∗∗∗^	0.38^∗∗∗^	0.72^∗∗∗^	-				
**Time 1 Emotional insecurity**												
5. Involvement in conflict	2.00	9.00	4.77	1.66	0.18^∗^	0.14	0.31^∗∗∗^	0.34^∗∗∗^	-			
6. Emotional reactivity	2.00	7.78	4.33	1.32	0.28^∗∗∗^	0.15	0.48^∗∗∗^	0.40^∗∗∗^	0.47^∗∗∗^	-		
7. Avoidance of conflict	2.00	6.25	3.12	0.99	0.33^∗∗∗^	0.28^∗∗∗^	0.43^∗∗∗^	0.45^∗∗∗^	0.16^∗^	0.53^∗∗∗^	-	
**Time 2 Inhibitory conflict**												
8. Inhibitory control	6.38	13.62	10.71	1.21	−0.19^∗^	−0.21^∗∗^	−0.21^∗∗^	−0.25^∗∗^	0.06	−0.03	−0.25^∗∗^	-

∗ *p* < 0.05;

∗∗ *p* < 0.01;

∗∗∗ *p* < 0.001.

### Model 1: Regressing Interparental Conflict on Inhibitory Control

The model in which the interparental conflict at Time 1 was regressed on the inhibitory control of children at Time 2 fits the data well [*χ*^2^(8) = 4.24, *p* = 0.84; *χ*^2^/*df* = 0.53; CFI = 1.00; RMSEA = 0.00; and SRMR = 0.03]. An estimated path between interparental conflict and inhibitory control was significant, which indicates that the interparental conflict was a significant predictor of inhibitory control (*β* = −0.29 and *p* < 0.01). That is, higher levels of interparental conflict were associated with lower levels of the children’s inhibitory control 6 months later.

### Model 2: Emotional Insecurity as a Mediator

**Figure 1:**
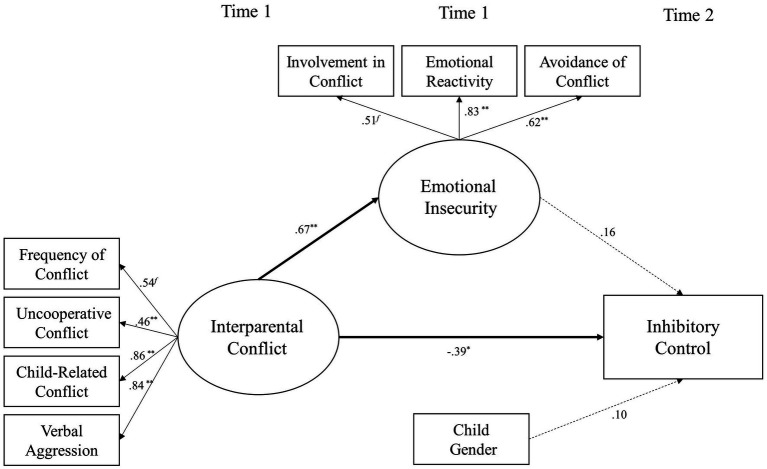
A structural equation model examining emotional insecurity of children as a mediator linking destructive interparental conflict to inhibitory control of children. ^∗^*p* < 0.05 and ^∗∗^*p* < 0.01.

The latent variable, emotional insecurity, was added to the previous model as a mediator of the relationship between interparental conflict and inhibitory control (shown in Figure 1). This model fits the data well [*χ*^2^(24) = 43.05, *p* < 0.05; *χ*^2^/*df* = 1.79; CFI = 0.95; RMSEA = 0.07; and SRMR = 0.06]. Interparental conflict at Time 1 was positively related to the emotional insecurity of children at Time 1 (*β* = 0.67 and *p* < 0.01). Interparental conflict at Time 1 was negatively related to the inhibitory control of children at Time 2 (*β* = −0.39 and *p* < 0.05). However, the emotional insecurity of children at Time 1 was not significantly related to their inhibitory control at Time 2 (*β* = 0.16 and *p* > 0.05). The bias-corrected bootstrap CI method was used to test the significance of the mediated relationship between interparental conflict and the inhibitory control of children *via* emotional insecurity (MacKinnon et al., 2004). Based on 5,000 bootstrap samples, there was no indirect effect (β = 0.11, *SE* = 0.16, 95% CI [−0.12, 0.48]).

### Model 3a: Involvement as a Mediator

**Figure 2:**
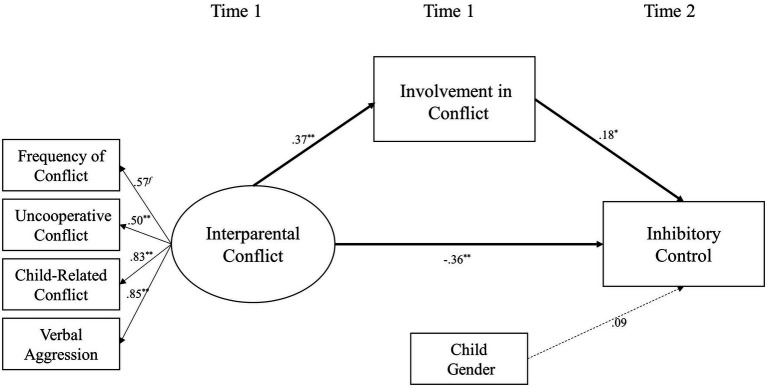
A structural equation model examining involvement of children as a mediator linking destructive interparental conflict to inhibitory control of children. ^∗^*p* < 0.05 and ^∗∗^*p* < 0.01.

The first dimension of emotional insecurity – involvement – was added to Model 1 as a mediator of the relationship between interparental conflict and inhibitory control (shown in Figure 2). This model also fits the data well [*χ*^2^(13) = 20.56, *p* = 0.08; *χ*^2^/*df* = 1.58; CFI = 0.97; RMSEA = 0.06; SRMR = 0.05]. Interparental conflict at Time 1 was positively related to the involvement of children at Time 1 (*β* = 0.37 and *p* < 0.01) and negatively related to inhibitory control at Time 2 (*β* = −0.36 and *p* < 0.01). The involvement of children at Time 1 was positively related to their inhibitory control at Time 2 (*β* = 0.18 and *p* < 0.05). Mediation analyses indicated that the involvement of children marginally mediated the relationship between interparental conflict and inhibitory control (*β* = 0.07, *SE* = 0.04, 95% CI [0.01, 0.16]).

### Model 3b: Emotional Reactivity as a Mediator

**Figure 3:**
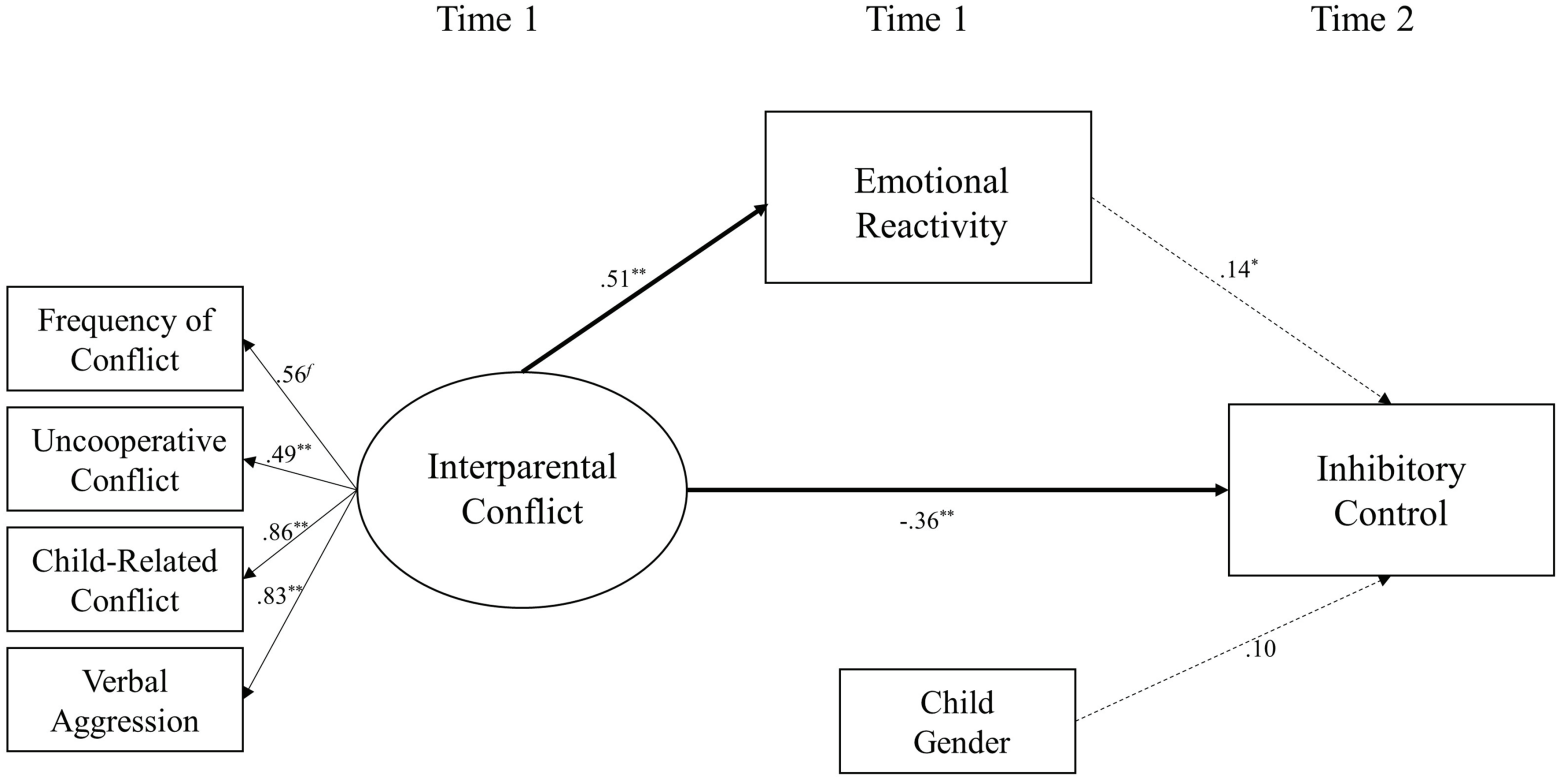
A structural equation model examining emotional reactivity of children as a mediator linking destructive interparental conflict to inhibitory control of children. ^∗^*p* < 0.05 and ^∗∗^*p* < 0.01.

The second dimension of emotional insecurity – emotional reactivity – was added to Model 1 as a mediator of the relationship between interparental conflict and inhibitory control (shown in Figure 3). This model also fits the data well [*χ*^2^(13) = 23.48, *p* < 0.05; *χ*^2^/*df* = 1.81; CFI = 0.96; RMSEA = 0.07; and SRMR = 0.05]. Interparental conflict at Time 1 was positively related to the emotional reactivity of children at Time 1 (*β* = 0.51 and *p* < 0.01) and negatively related to inhibitory control at Time 2 (*β* = −0.36, *p* < 0.01). The emotional reactivity of children at Time 1 was not significantly related to their inhibitory control at Time 2 (*β* = 0.14 and *p* > 0.05). Mediation analyses indicated that the emotional reactivity of children did not mediate the relationship between interparental conflict and inhibitory control (*β* = 0.07, *SE* = 0.06, 95% CI [−0.02, 0.19]).

### Model 3c: Avoidance as a Mediator

**Figure 4:**
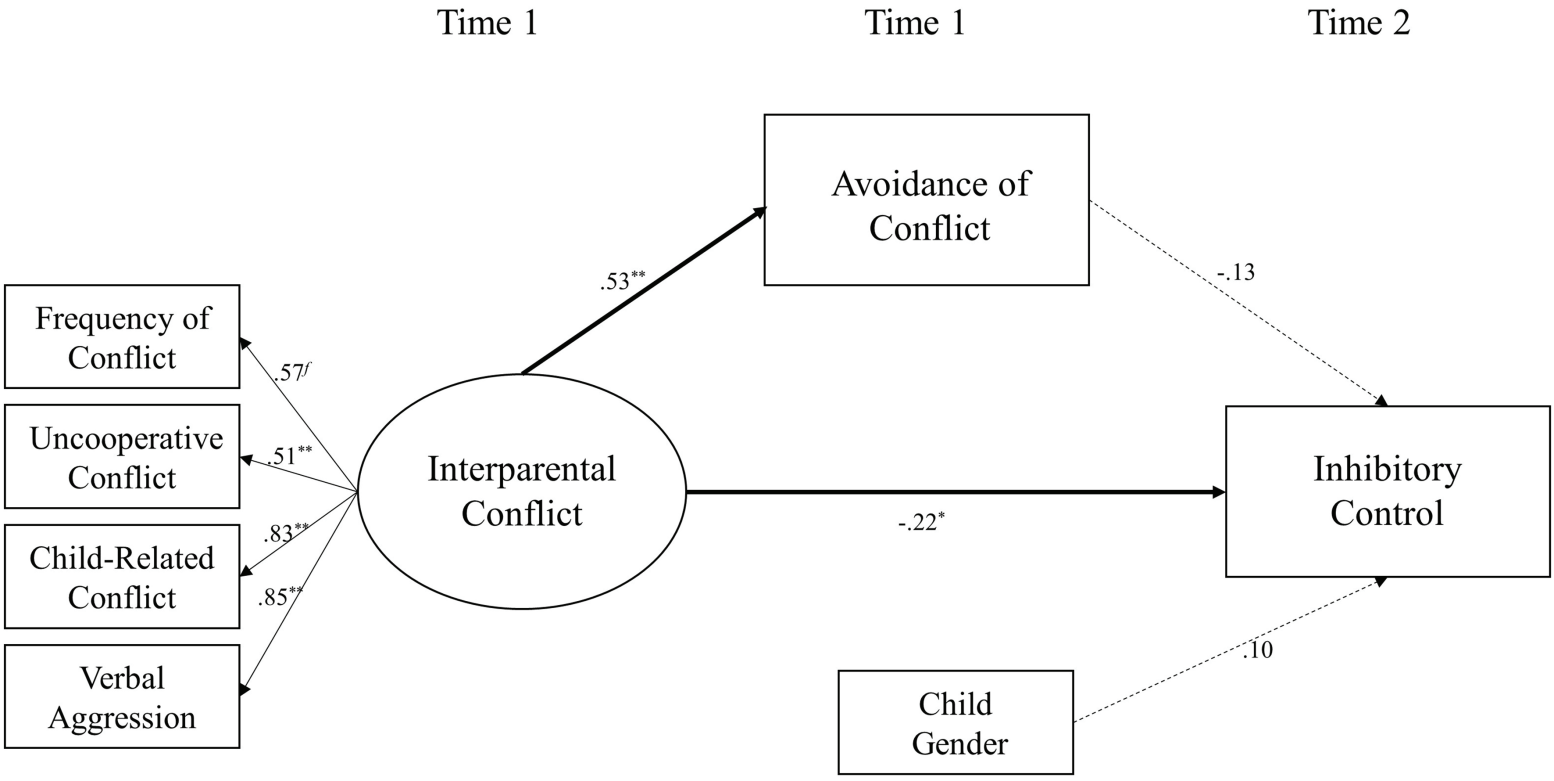
A structural equation model examining avoidance of children as a mediator linking destructive interparental conflict to inhibitory control of children. ^∗^*p* < 0.05 and ^∗∗^*p* < 0.01.

The third dimension of emotional insecurity – avoidance – was added to Model 1 as a mediator of the relationship between interparental conflict and inhibitory control (shown in Figure 4). This model also fits the data well [*χ*^2^(13) = 18.36, *p* = 0.14; *χ*^2^/*df* = 1.41; CFI = 0.98; RMSEA = 0.05; and SRMR = 0.04]. Interparental conflict at Time 1 was positively related to the avoidance of children at Time 1 (*β* = 0.53 and *p* < 0.01) and negatively related to inhibitory control at Time 2 (*β* = −0.22 and *p* < 0.05). The avoidance of children at Time 1 was not significantly related to their inhibitory control at Time 2 (*β* = −0.13 and *p* > 0.05). Mediation analyses indicated that the children’s avoidance did not mediate the relationship between interparental conflict and inhibitory control (*β* = −0.07, *SE* = 0.06, 95% CI [−0.18, 0.05]).

## Discussion

Although many studies have focused on the impact of interparental conflict on the adjustment problems of children (Cummings and Davies, 2010), few have examined its influence on the executive functioning of children, such as inhibitory control. Using a two-wave design with a Korean sample, this study investigated the link between interparental conflict and inhibitory control of children. When the gender of children was controlled, the results indicated that there were negative effects of interparental conflict on the inhibitory control of children 6 months later. These results are broadly consistent with social learning theory, which highlights that family is the context for learning behavior that is related to regulating impulsive responses (e.g., Davies et al., 2002b; Abbassi and Aslinia, 2010). Interpreted within this framework, children could observe the difficulties of their parents in controlling their emotions and behaviors in a destructive conflict context, and they could fail to learn effective regulation behaviors from their parents.

This study also anticipated that the emotional insecurity of children, which was a latent variable, would mediate the association between interparental conflict and their inhibitory control 6 months later. However, our findings did not support the mediating effect of the emotional insecurity of children on this relationship. Links between interparental conflict and inhibitory control and between interparental conflict and emotional insecurity were established, but emotional insecurity was not a significant predictor of inhibitory control. Additionally, this study separately examined the mediating effects of the three observational variables of emotional insecurity on the relationship between interparental conflict and inhibitory control. Interestingly, involvement mediated the association between interparental conflict and inhibitory control, while emotional reactivity and avoidance did not.

We propose several possible reasons for our results. First, all of the observational variables (involvement, emotional reactivity, and avoidance) are behaviors that children can exhibit in emotionally insecure situations during the interparental conflict. However, children may show different behaviors depending on their sensory processing patterns, which may have different effects on the inhibitory control of children. In Model 3a, involvement partially mediated the association between interparental conflict and inhibitory control. Interparental conflict expanded the involvement behaviors of children during the conflict and, in turn, increased inhibitory control. This result may be particularly relevant to one of the two hyposensitive patterns in Serafini et al. (2017), in which individuals engage in rich sensory activities. In this hyposensitive pattern, when exposed to sensory stimuli (e.g., interparental conflict in this study), an individual is resilient to impulsivity (e.g., inhibitory control). According to Serafini et al. (2017), an individual with the other type of hyposensitive pattern or with the hypersensitive pattern fails to detect a sensation or experiences discomfort with sensory stimuli, respectively, thereby showing greater impulsivity. The overt emotional reactivity or avoidance during the interparental conflict that is observed in some children may be related to the aforementioned sensory-failing hyposensitive‐ and hypersensitive-patterns. Psychiatric researchers considered the hyposensitive pattern of sensory seeking to be a protective and resilient trait that facilitates physical and social interactions and helps to create a resilience-promoting environment (Masten, 2007; Engel-Yeger et al., 2016). Although the involvement behaviors of children during the interparental conflict may be closely related to the hyposensitive pattern of sensory seeking, the SIMS-PR that was used to identify emotional insecurity in this study does not reflect the sensory processing patterns. Future studies need to focus on clarifying the role of sensory processing patterns in emotional security processes of children.

Second, the positive effects of the involvement behaviors of children during the interparental conflict on inhibitory control may be due to the nature of the study sample. According to Oh et al. (2011), Korean children try to help to resolve interparental conflict and directly intervene when the conflict is less intense and child-related. Most participants in this study were not in danger of experiencing serious and persistent interparental conflict as they were married and cohabiting couples from affluent families with a higher socioeconomic status and education level. Therefore, children in such families are more likely to attempt to regulate interparental conflict with relatively mild distress. Children who interfered in the conflict would have activated an inhibitory control function to defend against additional threats that may arise in interpersonal relationships. Considering Korean Confucianism culture, the children may well have followed the instructions of parents for children to control their behavior to achieve family cohesion and harmony.

However, these interpretations should focus on the following results. The positive influence of involvement on inhibitory control was not sufficient to overturn the negative effect of interparental conflict on inhibitory control. There are developmental advantages and disadvantages to the involvement reactions of children during the interparental conflict (Davies et al., 2016b). On the one hand, desirably, it can develop an openness to intimacy, empathic orientation, control of behavior of children in a developmental context, and participation in interpersonal relationships. On the other hand, an acute awareness of the threatening implications of conflict, perseverance in interpersonal relationships in the family, and constant emotional control can increase actual anxiety. In this study, the period between Times 1 and 2 was 6 months, which is a short period to examine the accumulated influence of involvement. Therefore, future studies need to investigate how involvement affects inhibitory control when it lasts for a long time.

Third, in Models 3b and 3c, emotional reactivity and avoidance did not significantly predict inhibitory control. In the models, emotional reactivity refers to the expressions of intense, prolonged, and dysregulated bouts of distress in children, and avoidance involves the strategies used by the children to avoid or escape interparental conflict or its adverse aftermath (Davies et al., 2002a). In this study, the emotional reactivity of children and avoidance may belong to the demobilizing pattern proposed in the reformulation of emotional security theory (EST-R; Davies et al., 2016b). Unlike the original version of the EST, the EST-R does not consider various reactions of children to interparental conflict as a single concept. Instead, their reactions are divided into various social defense patterns that limit exposure to interpersonal threats (i.e., secure, mobilizing, dominant, and demobilizing patterns; Davies et al., 2016b). According to the EST-R, demobilizing patterns can take a wide array of forms, ranging from high levels of arousal (e.g., freezing, vigilance, and gingerly moving away) to low levels of arousal (e.g., sadness, helplessness, fatigue, and postural slump). In addition, children who adopt the demobilizing strategy are more likely to develop internalizing symptoms, such as depression or anxiety. In this study, inhibitory control focused on the cognitive regulation of external behavior rather than emotional regulation. Therefore, emotional reactivity and avoidance may not be associated with inhibitory control, which cognitively controls external behavior, after 6 months.

The following limitations must be considered in order to accurately interpret the results for further studies. First, the major study variables were measured using parental reports, which have the following advantages. Parental reports have converged with observational methods in previous research (Kochanska et al., 2000), and they allow parents to observe behaviors of their children in a variety of situations beyond the laboratory context as interparental conflict cannot be easily be induced in a laboratory setting for ethical reasons. Studies of interparental conflict that used the CPS have found a stronger association between interparental conflict and child outcomes than studies that have employed other methods of assessing physical violence (Kitzmann et al., 2003). However, parental reports of emotional security of children do not capture all emotional experiences. Given the fact that the perceptions of parents of the temperament of their children and parent–child interaction patterns can potentially influence maternal ratings of the behaviors of children, the lack of observational measures of the research variables is a limitation of this study. Additionally, parental reports of the frequency of interparental conflict cannot accurately indicate the amount of the exposure of children to interparental conflict. Although confining our sample to cohabiting parents may have ameliorated this problem to some extent, the inability to accurately measure the extent of the exposure of children to conflict and the lack of observational measures of emotional security are another limitations of this study. Therefore, future studies should supplement this methodology as a countermeasure to capture subjective experiences of children when they are exposed to interparental conflict.

Second, the study variables were not measured repeatedly each time, and only one covariate (i.e., the gender of the child) was included, although this study did employ a two-wave SEM design with a Korean sample. As a result, the current findings do not fully explain the potential transactional processes among interparental conflict, emotional insecurity of children, and inhibitory control. Future studies need to consider repeated measures and other possible covariates, such as the temperament of children, sensory processing patterns, working memory, and early psychological problems.

Lastly, our participants were married and cohabiting couples from middle‐ and high-income families, who displayed little change in family relations. Therefore, the findings may not be generalizable to other samples (e.g., low-income families and clinical samples of children). Thus, future studies should involve a sufficient number of participants with multiple social backgrounds.

Despite these limitations, the results of our study that used a short-term longitudinal design helped us to advance our understanding of the association between interparental conflict and inhibitory control of children in Korean families. The findings highlight the need to examine executive functioning of children as well as the various dimensions of the reactions of children to interparental conflict. The role of emotional security in the connection between interparental conflict and the inhibitory control of children was interpreted by considering sensory processing patterns, the EST-R, and the influence of Confucianism on Korean culture.

## Data Availability Statement

The original contributions presented in the study are included in the article/supplementary material, further inquiries can be directed to the corresponding author.

## Ethics Statement

The studies involving human participants were reviewed and approved by Kyung Hee University IRB. Written informed consent to participate in this study was provided by the participants’ legal guardian/next of kin.

## Author Contributions

Y-EL was a primary investigator. SS was a corresponding author for this study. All authors contributed to the article and approved the submitted version.

### Conflict of Interest

The authors declare that the research was conducted in the absence of any commercial or financial relationships that could be construed as a potential conflict of interest.
